# Effects of Traditional Processing Techniques on the Nutritional and Microbiological Quality of Four Edible Insect Species Used for Food and Feed in East Africa

**DOI:** 10.3390/foods9050574

**Published:** 2020-05-04

**Authors:** Dorothy N. Nyangena, Christopher Mutungi, Samuel Imathiu, John Kinyuru, Hippolyte Affognon, Sunday Ekesi, Dorothy Nakimbugwe, Komi K. M. Fiaboe

**Affiliations:** 1 Department of Food Science and Technology, Jomo Kenyatta University of Agriculture and Technology, Nairobi 00100, Kenya; dorothynyangena@gmail.com (D.N.N.); samuel.imathiu@jkuat.ac.ke (S.I.); jkinyuru@agr.jkuat.ac.ke (J.K.); 2 International Institute of Tropical Agriculture (IITA), Mikocheni Light Industrial Area, Dar es Salaam 34441, Tanzania; 3 West and Central African Council for Agricultural Research for Development (CORAF), Dakar 3120, Senegal; h.affognon@coraf.org; 4 International Centre of Insect Physiology and Ecology, Nairobi 00100, Kenya; sekesi@icipe.org; 5 Department of Food Technology and Nutrition, School of Technology, Nutrition and Bio-Engineering, Makerere University, Kampala 7062, Uganda; dnakimbugwe@gmail.com; 6 International Institute of Tropical Agriculture (IITA), Yaoundé 2008, Cameroon; k.fiaboe@cgiar.org

**Keywords:** entomophagy, processing, traditional knowledge, food/feed safety, nutrition

## Abstract

Edible insects are increasingly being considered as food and feed ingredients because of their rich nutrient content. Already, edible insect farming has taken-off in Africa, but quality and safety concerns call for simple, actionable hazard control mechanisms. We examined the effects of traditional processing techniques—boiling, toasting, solar-drying, oven-drying, boiling + oven-drying, boiling + solar-drying, toasting + oven-drying, toasting + solar-drying—on the proximate composition and microbiological quality of adult *Acheta domesticus* and *Ruspolia differens*, the prepupae of *Hermetia illucens* and 5th instar larvae of *Spodoptera littoralis*. Boiling, toasting, and drying decreased the dry matter crude fat by 0.8–51% in the order: toasting > boiling > oven-drying > solar-drying, whereas the protein contents increased by 1.2–22% following the same order. Boiling and toasting decreased aerobic mesophilic bacterial populations, lowered *Staphylococcus aureus*, and eliminated the yeasts and moulds, Lac+ enteric bacteria, and *Salmonella.* Oven-drying alone marginally lowered bacterial populations as well as yeast and moulds, whereas solar-drying alone had no effect on these parameters. Oven-drying of the boiled or toasted products increased the aerobic mesophilic bacteria counts but the products remained negative on Lac+ enteric bacteria and *Salmonella*. Traditional processing improves microbial safety but alters the nutritional value. Species- and treatment-specific patterns exist.

## 1. Introduction

Insects are part of the diets of humans and domesticated animals in many parts of the world. They have been consumed by communities for many years, and were suggested as a resource that could be used to ease global food shortages [1]. It has been claimed that insects provide vital nutrients including protein, calories, minerals and useful bioactive compounds to more than 2 billion people worldwide [2]. In Africa, insects contribute to the livelihoods and food security of households by generating significant income, and creating employment for the local communities [3]. Edible insect species that are considered unsuitable for consumption by humans have also been used as ingredients to substitute conventional protein sources, e.g., fishmeal in poultry, fish and pig feeds, thus contributing indirectly to human diets [4,5,6]. Already in East Africa, edible insect farming initiatives have taken off, and some countries have developed regulatory mechanisms to mainstream their production and use, for example, in animal feeds [7]. Hence, there are opportunities for farmers to produce edible insects, which can be delivered to food and feed factories as a raw material for manufacturing value-added products.

To be able to utilize edible insects on a commercial scale, the production of large quantities of biomass is necessary, either through mass rearing or sustainable harvesting. However, the need for suitable postharvest techniques that can make it possible to accumulate practical quantities that are of high quality is also important. Notwithstanding the fact that many insect species are collected from wild habitats or likely to be reared in environments that potentially originate microbiological hazards [8,9,10,11], the rich nutritional profile of insects offers a suitable substrate for the growth of unwanted microorganisms, such as spoilage and pathogenic ones, when the conditions are suitable [12,13]. In Africa, freshly harvested or semi-processed insects find their way to rural open-air markets, with some favourite species consumed by humans reaching urban markets and restaurants [3,14]. The processes leading to delivery of the edible insects to the end-consumer are highly variable, as the techniques and practices in collection or harvesting, aggregation, handling, preliminary processing, packaging, storage, and transportation vary widely.

The harvesting of microbiologically hazard-free insects is difficult to achieve. The contamination of insects with unwanted microorganisms is a consequence of a combination of the substrates, the insect species, and the farming or collection environments and processing steps applied [10]. Wynants [15] examined the microbial dynamics during an industrial production cycle of lesser mealworms (*Alphitobius diaperinus*), and found a direct association between the microbial diversity of the substrate and the harvested insects. The microbial populations were, however, generally lower in the larvae compared to the substrate, feed remnants, faeces and exuviae. In a separate study, the microbial dynamics during the industrial rearing, processing, and storage of the edible house cricket, *Gryllodes sigillatus*, were reported [16]. The microbial diversity of the feed substrate and harvested crickets were similar. However, unlike *A. diaperinus*, the overall microbial population was higher in the crickets. In both studies, food pathogens including *Salmonella* spp., *Listeria monocytogenes*, *Bacillus cereus* or coagulase-positive staphylococci were not detected, but fungal isolates corresponding to the genera *Aspergillus* and *Fusarium* were recovered. Elsewhere [11], the possibility of transmission of *Salmonella* sp. to mealworms (*Tenebrio molitor*) reared on contaminated wheat bran substrate was investigated. Survival of *Salmonella* sp. in the larvae was dependent on the contamination level with little or no retention at the lower levels, possibly because of competitive exclusion by the endogenous larval microbiota and/or because of antibacterial activity of the larvae. These findings indicated that some bacterial species have a competitive advantage and become dominant depending on the insect species. Regardless of these complex dynamics, processing presents a critical line of defence against potential hazards. It also interrupts spoilage processes, thereby improving product quality and minimizing product losses. For this reason, recent reviews have concluded that there is a need to upgrade and standardize processing methods so that the safety and nutritional value of insect-based food/feeds can be assured [10,17,18,19].

Some popular processing methods of edible insects in Africa (see Supplementary Material S1) include steaming, boiling, roasting, toasting, frying, smoking, and drying, or a combination of these [17]. A significant reduction in microbial hazards can be achieved by approaches such as thermal treatment, but some procedures may fail to adequately achieve the desired results [12]. The evaluation and validation of these methods that build on traditional knowledge would be a strong entry point for developing and implementing a successful food/feed safety mechanism, especially in rural settings where insect rearing and collection can have the greatest impacts in securing livelihoods. Thus, the aim of the present work was to examine the effect of some popular processing methods on the nutritional and microbiological quality of different insect species, Specifically, we asked: (i) how do processing techniques affect the nutritional value and microbiological quality? (ii) for the same processing technique, does the nutritional value, and microbiological quality of the processed products vary with the insect species? (iii) is there interaction between processing technique and insect species? Such knowledge would contribute crucial guidance for technological improvements in hazard control plans targeting rural collectors and the small-scale actors involved in insect rearing and preliminary processing activities.

## 2. Materials and Methods

### 2.1. Sources of Edible Insect Samples

Adult house crickets (*Acheta domesticus*) reared on a mixture of brewer’s waste and kales, black soldier fly (*Hermetia illucens*) pre-pupae reared on a mixture of brewer’s and kitchen waste, and African cotton leafworm (*Spodoptera littoralis)* 5th instar larvae reared on black nightshade leaves, were collected from the insect rearing and containment unit of the International Centre for Insect Physiology and Ecology (ICIPE) in Nairobi, Kenya. Adult grasshoppers (*Ruspolia differens*) were collected from the wild in Kampala Central, Nakawa and Makidye divisions, Uganda, and transported overnight in cool boxes to Jomo Kenyatta University of Agriculture and Technology (JKUAT, Nairobi, Kenya), Food Science laboratory for analysis. All the raw insects were washed with chilled tap-water (4 °C) before any processing. All samples were sieved and separated from undesired debris at the point of collection. The samples for microbiological analysis were analysed within 24 h of collection or processing, during which period they were stored in a refrigerator maintained at 4–8 °C. Samples for chemical analysis were preserved in a deep freezer (−21 °C) in sterile zip-lock bags.

### 2.2. Experimental Design

A factorial design of two factors—insect species and processing method—was applied. There were four levels of insect species—*A. domesticus*, *R. differens*, *H. illucens*, *S. littoralis*—and nine levels of processing method—toasting, boiling, solar-drying, oven-drying, toasting + solar-drying, toasting + oven-drying, boiling + solar-drying, boiling + oven-drying—and finally, the raw insects washed in chilled water. The experiment was replicated three times.

### 2.3. Post-harvest Processing

#### 2.3.1. Toasting

A clean, dry, stainless pan was placed over an open flame and heated to about 150 °C. The raw insects (500 g) were then placed on the hot pan and without addition of cooking oil fried for 5 min, with regular turning using a wooden cooking stick to avoid sticking or burning [20]. The toasted products were then transferred on to an aluminium foil and left for 20 min to equilibrate to room temperature (22–25 °C). The product was then subdivided into two lots which were packed separately in polyethylene zip-lock bags and stored in a refrigerator or deep freezer awaiting microbiological and chemical analysis, respectively.

#### 2.3.2. Boiling

The raw insects (500 g) were placed on a wire-mesh kitchen sieve and submerged in a boiling water bath (96 °C) for 5 min [12]. The sieve was then lifted from the water, and the contents were allowed to drain for 1 min. The insects were transferred on to an aluminium foil and left for 20 min cool to room temperature (22–25 °C). The product was then subdivided into two lots which were then packed in polyethylene zip-lock bags and stored in a refrigerator or freezer awaiting microbiological and chemical analysis, respectively.

#### 2.3.3. Solar-Drying and Oven Drying

For the solar-dried products, the raw insects (500 g) were placed in a solar dryer, and left to dry to constant weight in 2–3 days. Details of the dyer design were as described elsewhere [21]. Briefly, the design consisted of a clear plastic (polyethylene) sheet stretched over a wooden box (0.6 m wide × 1.2 m long × 0.2 m high), which was placed longitudinally on a slanting metal frame constructed to a height of 1 m of the ground on the air inlet end, and 1.2 m on the air exit end. The inside of the box was lined with a black polyethylene sheet, and the air entry and exit ends were drilled with closely spaced holes of 1 cm diameter. The temperature and relative humidity in the dryer before introducing the insects ranged between 50–60 °C and 15–25%, respectively, as determined using an EL-USB-2 data logger (Lascar electronics Inc., Erie, PA, USA). For the oven-dried samples, raw insects (500 g) were placed in an air-oven dryer (TD-384KN model Thermotec, Tokyo, Japan), maintained at 60 °C and dried to constant weight in 2–3 days. The samples were regarded dry when the change in weight was less than 1% over three samplings performed at one-hour intervals. The products were removed from the drying chambers and left to cool for 20 min, following which they were subdivided into two lots, which were then packed in polyethylene zip-lock bags and stored in a refrigerator or freezer awaiting microbiological and chemical analysis, respectively.

#### 2.3.4. Combined Processes (Boiling/Toasting and Drying)

Separate lots (500 g) of raw insects were toasted or boiled as described in Section 2.3.1 and Section 2.3.2, respectively. These were then dried either in the solar-dryer or in the oven-dryer, as described in Section 2.3.3. Each of the final products was then subdivided into two lots, which were then packed in polyethylene zip-lock bags and stored in a refrigerator or freezer awaiting microbiological and chemical analysis, respectively.

### 2.4. Determination of Proximate Composition

The AOAC standard methods [22] were used. Moisture content was determined by hot-air drying (Method 925.10), crude protein by semi-micro Kjeldahl method for total nitrogen with 6.25 as the nitrogen-to-protein conversion factor (Method 920.87), crude fat by soxhlet extraction (Method 920.85), crude fibre by the Henneberg–Stohmann method (Method 920.86), and crude ash by incineration in a muffle furnace (Method 923.03). Total available carbohydrate was determined by the difference.

### 2.5. Assessment of Microbiological Quality

#### 2.5.1. Enumeration of Total Viable Count, Lactose Positive Enteric (Lac+) bacteria, and *Staphylococcus Aureus*

The raw and processed products were each separately pulverized in a Philips HR-2850 mini blender that was pre-rinsed with 70% ethanol. Ten (10) grams of the pulverized material was transferred into a conical flask containing 90 mL of sterile peptone water (HiMedia M028) as a diluent, and the mixture was homogenized in a sterile polyethylene bag using a Stomacher^®^ 400 Circulator (Seward, West Sussex, UK) for 2 min. Aliquots (1 mL) of the homogenate were serially diluted up to 10^−7^ in 9 mL of the diluent. Aliquots (0.1 mL) of individual dilutions were cultured in triplicate Petri dishes. Total viable counts (TVC) were determined by pour-plate technique on Plate Count Agar (HiMedia M091) after incubation at 35 °C for 48 h. The TVC provided an estimate of the total viable aerobic mesophilic microbial population present in the samples. Lac+ enteric bacteria were determined on MacConkey Agar (HiMedia M081) following 24 h incubation of the cultured plates at 37 °C. Typical colonies were distinguished by their pink-red colour. *Staphylococcus aureus* counts were determined on Baird Parker Agar (HiMedia M043) containing 5% Egg Yolk Tellurite Emulsion (HiMedia FD046) following incubation of the cultured plates at 35 °C for 48 h. Typical colonies were distinguished by their circular (2–3 mm diameter), smooth, convex, moist appearance, and confirmed using coagulase test. The latter was performed by emulsifying a portion of isolated colonies in physiological saline on a microscope slide to form a suspension, then mixing with rabbit plasma and examining the clumping behaviour of the cells. For all the determinations, plates having 20–300 colonies were counted, and the number was expressed as log-colony-forming units per gram (Log CFU/g) of sample.

#### 2.5.2. Enumeration of Yeasts and Moulds

The determination of total yeasts and mould counts was performed on Potato Dextrose Agar (PDA) (HiMedia Ref. M096) acidified with sterile 10% tartaric Acid (251380, Sigma-Aldrich, Schnelldorf, Germany) to pH 3.5. Aliquots of sample homogenate prepared as in Section 2.5.1 above were serially diluted. Aliquots (0.1 mL) of individual dilutions were cultured in triplicate PDA plates using the spread plate technique, and the plates incubated at 25 °C for 72 h. Plates having 20–300 colonies were counted, and the number expressed as log-colony-forming units per gram (Log CFU/g) of sample.

#### 2.5.3. Detection of Salmonella

Samples (25 g) of the raw and processed products were enriched with 225 mL nutrient broth containing 5 g peptone, 5 g sodium chloride, 1.5 g beef extract, and 1.5 g yeast extract per 1000 mL of water, pH 7.4 (HiMedia M002) and incubated at 35 °C for 24 h. Selective enrichment was then performed by transferring 25 mL of the enriched homogenate to 225 mL of tetrathionate broth (HiMedia M032) and the culture incubated at 37 °C for 24 h. A loopful of the Tetrathionate broth culture was streaked on Salmonella-Shigella Agar (HiMedia, M108) and plates incubated at 37 °C for 24 h. Typical *Salmonella* colonies (colourless colonies with black centres) were further identified using the Triple Sugar Iron (HiMedia, M021I) test. For this test, a sterilized inoculation needle was used to touch the top of a well isolated colony and then inoculated into the Triple Sugar Iron agar by first stabbing through the centre of the media to the bottom of the tube, followed by streaking on the surface of the agar slant. The slants were incubated at 35 °C for 24 h. *Salmonella* forms a red slope (alkaline) and yellow (acid) butt indicating fermentation of glucose, with or without gas (cracks and bubbles in the medium), and with or without H_2_S production (blackening of the medium).

### 2.6. Statistical Analyses

Data were subjected to analysis of variance (ANOVA) using general linear model (GLM) procedures on IBM^®^ SPSS^®^ Statistics 20 (IBM Corporation, Armonk, NY, USA). Insect species and processing method were the two independent factors, while proximate composition parameters (moisture, crude protein, crude fat, crude fibre, ash, total carbohydrate contents) and microbiological parameters (total viable count, and counts of Lac+ bacteria, *St. aureus*, yeasts and moulds) were the dependent variables. For the experimental part examining the effects of processing on proximate composition, five factor levels of processing technique were considered (toasting, boiling, oven-drying, solar-drying, and the raw insects after washing in chilled water), whereas all nine levels were considered for the experimental part designed to examine the effects on microbiological quality. When interactions between insect species and processing method were significant, a one-way ANOVA was performed to test the significance of differences amongst insect species and processing method combinations. Means were separated using Bonferroni’s *t*-test at *p* < 0.05.

## 3. Results and Discussion

### 3.1. Proximate Composition of Raw Insects

Dry matter (DM) contents of the fresh *A. domesticus*, *R. differens*, *H. illucens* prepupae, and *S. littoralis* larvae ranged between 26–35% (Table 1). The *A. domesticus* and *R. differens* had higher DM contents than *H. illucens* and *S. littoralis* larvae. The dry matter crude protein and crude fat also differed significantly (see Table 1 and Table 2). Protein contents followed the order *A. domesticus > R. differens* > *H. illucens* > *S. littoralis*. Except for *S. littoralis*, the protein contents were within the known ranges in the literature: 55–70%, 34.2–45.8%, 38–48% for *A. domesticus* [23], *R. differens* [24], and *H. illucens* [4], respectively. The protein content of *S. littoralis* was lower by a factor of 0.75 compared to the 51% reported by others [25]. The huge difference may be attributed to the substrates on which the caterpillars were reared, i.e., castor bean (*Ricinus communis*) leaves, which are richer in protein compared to black nightshade leaves (*Solanum nigrum*) in our case. Of the four insects, *H. illucens* and *R. differens* contained more fat by a factor of 1.5 compared to *A. domesticus* and *S. littoralis* (see Table 1). Furthermore, the fat contents of *H. illucens* and *A. domesticus* were within the range reported by other researchers: 9.8–22.8% for *A. domesticus* [23] and 15–35% for *H. illucens* [4]. The fat contents of *R. differens* and *S. littoralis* were approximately half the levels reported elsewhere i.e., 42.2–54.3% for *R. differens* [24] and 33% for *S. littoralis* [25].

The fibre, ash and available carbohydrate contents of the raw insects are presented in Table 2. Crude fibre levels were generally <10%, and lowest in *R. differens*. The crude fibre contents of *R. differens*, *H. illucens*, and *S. littoralis* corresponded well with those reported by other authors i.e., *R differens*: 1.8–2.7% [24]; *H. illucens:* 7% [4]; *S. littoralis*: 10.7% [25], whereas the levels determined in *A. domesticus* were about half the levels (14.9–22.1%) reported elsewhere [23]. Crude fibre also includes chitin and complex carbohydrates such as cellulose and lignin that might be present in the insects’ gut [26], and therefore variability may arise depending on preparation processes such as the degutting, cleaning and removal of parts, or the inclusion of a starvation regime prior to harvesting [15]. Crude ash contents were about two times higher in *S. littoralis* compared to the other insects. The ash content of *R. differens* compared well with the findings reported elsewhere [20], whereas the ash content of *A. domesticus* was within the 3.6–9.1% range reported by other authors [23].

Previous studies show that edible insects are highly nutritious, but the nutritional composition varies widely. Protein, fat, fibre, and ash contents range between 4.9–77%, 0.7–77%, 0.9–29%, and 0.3–21% on DM basis, respectively [23]. The wide variability has been associated with insect species and feed substrates [27,28,29,30], collection sites and swarming seasons [24], age [31], sex [32], and possibly the use of varying methods or false interpretation. The insects examined in this study belonged to the orders Lepidoptera (*S. littoralis*), Orthoptera (*A. domesticus* and *R. differens*), and Diptera (*H. illucens*), and included adults (*A. domesticus* and *R. differens*), pre-pupae (*H. illucen*) and larvae (*S. Littoralis*). According to Rumpold and Schlüter [23], Orthopterans have, on average, a higher protein content (61.3%) compared to Dipterans (49.5%) and Lepidopterans (45.4%), whereas the Lepidopterans have a higher lipid content (27.7%) compared to Dipterans (22.8%) and Orthopterans (13.4%). Fibre contents are higher in Dipterans (13.6%) than Orthopterans (9.6%) and Lepidopterans (6.6%), while carbohydrates are higher in Lepidopterans (18.8%) compared to Orthopterans (13%) and Dipterans (6%). Ash content is higher in Dipterans (10.3%) than Lepidopterans (4.5%) and Orthopterans (3.9%). Examining our data within these categories, the findings on protein, fibre, and carbohydrate correspond well, while those on fat and ash do not, suggesting that the other factors (age, sex, feed substrates, collection sites and swarming seasons, etc.) also had strong effects. Age was reported to influence the nutritional composition of reared house crickets, with protein, total lipids and mineral content being highest at 9–12 weeks [31]. Regarding sex, females crickets were found to have more lipids and less proteins and chitin than the males [32]. *Hermetia illucens* larvae were shown to have the capacity to accumulate both lipid- and water-soluble nutrients from feed substrates [27]. Elsewhere, the *H. illucens* prepupae reared on digestate were low in lipids and high in ash compared to those reared on vegetable waste [29]. It was also reported that substrates comprising brewery waste or a mixture of fruit and vegetables resulted in higher protein content compared to fruit or winery by-product substrates [33]. Geographical area and swarming season were found to influence the nutritional composition of wild-harvested *R. differens* primarily due to differences in feed substrate, possibly arising from the type and abundance of available vegetation or soil type, which particularly influences the mineral composition of the vegetation [24].

### 3.2. Effects of Processing on Proximate Composition

The detailed statistical analysis results for effects of processing on proximate composition are presented in the Supplementary Material S2. The moisture contents of the processed products (Table 1) varied with the processing method (*F* = 628.7; df = 4; *p* < 0.001) and species (*F* = 28.3; df = 3; *p* < 0.001), and the interaction effect of processing method and insect species was significant (*F* = 92.1; df = 19; *p* < 0.001). Boiling resulted in moisture gain (10–18%) due to hydration or the increased binding of water by hydrophilic tissue components, whereas toasting resulted in a lower moisture content (30–60%) due to evaporative loss. A similar gain in moisture was reported in boiled *Gryllodes sigillatus* [16]. Drying reduced the moisture content of the raw insects by 85–90%, but oven-drying achieved lower moisture levels by about two percentage points compared to solar-drying. The *S. littoralis* gained more moisture during boiling, but also lost more moisture during toasting and drying, which indicates a deviation in hydration properties compared to the other insects. Earlier work [21] demonstrated dissimilarities in the hydration properties of *A. domesticus* and *H. illucens* powders, and attributed the differences to the compositional factors that influence the number and water binding strength of hydrophilic sites.

The crude protein contents increased by 1.2–22% relative to the raw product (see Table 1). The interaction of processing method and insect species was significant (*F* = 7.1; df = 19; *p* < 0.001), and the main effects were significant as well (processing: *F* = 99.4; df = 4; *p* < 0.001; insect species: *F* = 598.9; df = 3; *p* < 0.001). Boiling and toasting increased the protein contents remarkably (boiling: 8.9–11%; toasting 13–22%) on all the insects compared to solar-drying (1.5–3.5%) and oven-drying (1.2–7.1%). The increase in protein content upon drying was significant in *H. illucens* (both solar- and oven-drying) and in *A. domesticus* and *S. littoralis* (oven-drying) but not in *R. differens*. Other authors [34] reported a decrease in protein content when the beetle *Eulepida mashona* and the cricket *Henicus whellani* were boiled for 30–60 min (*E. Mashona*: 1.2–14.7%; *H. whellani* 9.5–10.1%), but then observed no change when the insects were toasted. The decrease was possibly due to the dissolution of protein or disintegration and loss of connective tissue as colloidal constituents in the boiling water. A decrease in crude protein content was also reported in studies with *Imbrasia belina* [34] and *Hemijana variegata* [35]. Other authors, e.g., [36], found no significant change in protein content when the edible caterpillar, *Imbrasia epimethea*, was thermally processed (boiled or boiled and sun-dried). A decrease in nitrogen content might also occur during thermal treatments due to loss of amides and amines [37] or the formation of complexes with primary and secondary lipid oxidation products [38]. Our results suggest that these effects were overshadowed by other dynamics such as the loss of dry matter components, mainly fat. In fact, the measured increase in protein content was strongly correlated with fat loss (*R^2^* = −0.692; *p* < 0.001). Solar-drying had minimal effect on protein content.

Processing decreased fat content by 2.0–51% (Table 1). Processing method and insect species were significant (processing method: *F* = 1209.1; df = 4; *p* < 0.001; insect species: *F* = 1734.9; df = 3; *p* < 0.001). Likewise, the interaction effect was significant (*F*= 55.5; df = 19; *p* < 0.001). Examining the main effects independently, toasting, boiling, oven-drying and solar-drying decreased the fat contents by 42.0%, 15.0%, 6.1% and 2.7%, respectively. Similarly, the loss of fat content followed the order *H. illucens* (24.2%) > *A domesticus* (15.3%) > *S. littoralis* (13.6%) > *R. differens* (12.8%). Fat loss correlated positively with the fat content of the raw insects (*R^2^* = 0.57; *p* <0.001). The average loss was also higher for the non-orthopterans, i.e., *H. illucens* pre-pupae and *S. littoralis* larvae (18.9%), compared to the orthopterans (14.1%), although the difference was not statistically significant (*p* = 0.326). The loss of fat during boiling was due to the melting of fat globules into the boiling water. Similarly, toasting melted the fat, which became exuded as a result of tissue contraction, while some of it was possibly also lost through thermal decomposition [39]. The decrease in fat content during oven- and solar-drying suggests that some fat may have transuded alongside water vapor or was oxidized into other compounds [40,41], especially since insect lipids are made up of polyunsaturated fatty acids. Furthermore, the lipids of different insect species comprise the fatty acid profiles of varied physico-chemical properties [42], which might explain some of the variability in fat loss magnitudes during thermal treatment.

Crude fibre (see Table 2) increased or decreased depending on processing method and insect species (*F* = 17.1; df = 19; *p* < 0.001). Except for *S*. *littoralis*, boiling diminished the contents (7.6–8.7%), whereas toasting increased them (2–22%). Similar results were reported with cooked and roasted mopane worms [26]. Heating shifts the ratio of soluble to insoluble fibre [43]. The decrease in fibre content during boiling was probably because some of the complex carbohydrates dissolved [44] or were washed off the tissues. Toasting, on the other hand concentrated these polysaccharides, especially with the concomitant loss of fat during the process. However, the thermal treatment may also have resulted in an increase in total fibre by causing the formation of protein–fibre complexes [43]. Unlike boiling and toasting, drying did not affect crude fibre contents.

The crude ash content of processed products (Table 2) was also a function of the interaction of processing method and insect species. The main effects were also significant. The ash contents generally decreased upon boiling due to leaching [34,45] but increased in the toasted products. We attribute this increase to concentration effects, unlike in the findings of others [26] where contamination from ash during hot-ash roasting was implicated. There was no significant change in ash contents when samples were subjected to drying, except for *R. differens*, which was peculiar. The contents of available carbohydrates (Table 2) were dependent on the interaction of insect species and processing (*F*= 26.3; df = 19; *p* < 0.001). The main effects were also significant (insect species: *F* = 460; df = 3; *p* < 0.001; processing method: *F*= 7.9; df = 4; *p* > 0.001). Nonetheless there were no clear trends, as the CHO determination was dependent on the increase or decrease in the other parameters. Generally, for all the proximate parameters, the interaction of insect species with processing method was significant, meaning that each combination of species and processing method produced a different outcome.

### 3.3. Effect of Processing on Microbial Quality

The detailed results of statistical analyses exploring effects of processing technique on microbial quality are presented in the Supplementary Material S3.

#### 3.3.1. Effects of Boiling and Toasting

Total viable count and YMC of the raw and processed products are presented in Table 3. The TVC determined on the raw insects (7.0–9.1 Log CFU/g) were comparable to those reported elsewhere [12,15,16]. The YMC (6.4–8.2 Log CFU/g) were higher by 2–3 log cycles compared to the YMC reported for industrially reared lesser mealworms (*Alphitobius diaperinus*) [15], but within the limits reported for industrially reared house crickets *Gryllodes sigillatus* [16]. The allowable TVC limit in edible foods is 7 Log CFU/g [46] and, therefore, all the raw insects except *S. littoralis* exceeded this limit. For animal feeds, however, the TVC would be within the allowable limit of 10^9^ CFU/g [7]. Similarly, the raw insects had a YMC higher than the recommended limit of 6 Log CFU/g [46].

Boiling and toasting lowered the TVC by 4–6 log cycles and completely eliminated the yeasts and moulds. Similar findings were reported for boiled (5–10 min) *G. sigillatus* [16], boiled (ca. 93 °C; 30 min) *I. belina* [47] and toasted (10 min) *R. differens* [48]. The interaction effect of insect species and processing method was significant for the destruction of TVC (*F* = 1207; df = 35; *p* < 0.001) and YMC (*F* = 11739.6; df = 35; *p* < 0.001). Furthermore, both main effects were significant for both TVC (processing method: *F* = 505.5; df = 8; *p* < 0.001; insect species: *F* = 10.26; df = 3; *p* < 0.001) and YMC (processing method: *F* = 526.1; df = 8; *p* < 0.001; insect species: *F* = 13.1; df = 3; *p* < 0.001). Boiling was more effective than toasting at lowering the TVC, due to better heat transfer through the tissues [12]. However, our results also show differences in the reduction in TVC and YMC across the insect species (see Table 3), which might be due to a combination of factors such as the initial contamination levels and size.

With the exception of *S*. *littoralis*, all the unprocessed insects were positive for Lac+ enteric bacteria (Table 4). This category comprises bacteria such as *Escherichia coli*, *Klebsiella*, and *Enterobacter* spp., and therefore may indicate contamination with faecal coliforms from unhygienic environments, soil or poor handling [49]. In dried insect products for compounding animal feeds, total coliform count should not exceed 500 CFU/g, while pathogenic *E. coli* should not exceeded the 10 CFU/g [7]. The absence of Lac+ enteric bacteria in *S*. *littoralis* was because of the hygienic conditions of rearing. Elsewhere, contamination with coliforms including *E. coli* was also reported in wild-harvested unprocessed emperor moth caterpillars [13,47] and grasshoppers [50]. The boiling and toasting procedures applied in the present study potentially eliminated *E. coli* and other enteric pathogens. Other authors, [47,48], made similar observations on boiled *I. belina* larvae and toasted *R. differens*, respectively.

*Staphylococcus aureus* was present on all the raw products (Table 5). The counts (7.7–9.1 Log CFU/g) were higher than the recommended limit of 4 Log CFU/g [46]. Habitat and manual collection might have been the cause of a significantly higher *St. aureus* contamination in the *R. differens* compared to the other insect species. Processing lowered the contamination levels by 0.9–4.5 Log CFU/g depending on the insect species and processing method (*F* = 3110.7; df = 35; *p* < 0.001). Boiling and toasting remarkably reduced *St. aureus* by 4.8–6.4 log cycles but unlike the Lac+ enteric bacteria, fungi, and *Salmonella*, complete elimination was not achieved. The results on *Salmonella* are shown in Table 5. Microbiological guidelines for food and feed require no detection of *Salmonella* in 25 g of sample [7,46,51]. All the raw insects were positive for *Salmonella*, but the products subjected to boiling or toasting tested negative; thus, boiling or toasting rendered the products safe.

#### 3.3.2. Effects of Solar- and Oven-Drying

Solar-drying did not affect TVC, while oven-drying lowered the counts by 0.5–2.3 log cycles depending on species (see Table 3). Likewise, drying only lowered the YMC in *H. illucens* and *A. domesticus* by one log cycle (solar) and 2.7–4.6 log cycles (oven). While yeasts are not known to cause food poisoning, strains of moulds that are capable of producing mycotoxins with injurious health consequences on humans and animal have been isolated in dried edible insect products [13,52]. Depending on insect species, solar-drying lowered Lac+ bacteria and *St. aureus* contamination by 3.3–4.3 and 3.9–5.4 log cycles, respectively (see Table 4 and Table 5). Nonetheless, this did not bring the contamination to the acceptable limits [46]. Contrastingly, oven-drying lowered the Lac+ bacteria and *St. aureus* contaminations by greater margins (Lac+ bacteria: 4.6–5.3 log cycles; *St. aureus*: 4.5–6.5 log cycles) bringing down the contamination levels to the acceptable limits. Both the oven- and solar-dried products, however, were positive for *Salmonella* (Table 5) and were therefore unsafe. We did not find works that investigated purely the effects of drying the raw insects on microbial contamination levels. However, some authors [53] investigated the effects of oven-heating on *E. coli*, and *St. aureus* contamination of beef chops and showed that these micro-organisms were destroyed at an internal cook temperature of 52–57 °C; the authors demonstrated D-values of 66.7, 4.4 and 0.47 min for *E. coli* and 61.4, 5.3 and 0.48 min for *St. aureus* at 52, 54 and 57 °C, respectively, suggesting that the destruction of these organisms could be expected when drying is done at temperatures close to 60 °C. Furthermore, the authors reported even lower D-values for *Salmonella* (15.3, 6.2, and 1.7 min at 52, 54 and 57 °C, respectively). The presence of *Salmonella* on the samples subjected to drying was probably due to the rather resistant nature of the pathogen to dry-heat [53] or recontamination.

#### 3.3.3. Effects of Combined Processing (Boiling or Toasting Followed by Solar- or Oven-Drying)

The products subjected to boiling or toasting followed by solar- or oven-drying were free of fungi, Lac+ bacteria and *Salmonella*, (See Table 3, Table 4, Table 5), and the *St. aureus* counts on the boiled or toasted and dried samples were lower. Thus, drying reduced the counts further, although marginally (see Table 5). The absence of fungi, *Salmonella* and coliforms including *E. coli* in boiled and dried *I. belina* was also reported, but recontamination, particularly by moulds and yeasts, was observed when the boiled products were subjected to open sun-drying [47]. Other authors reported the presence of *E. coli*, coliforms, and *Salmonella* on thermally processed and dried insects, for instance, on roasted (5 min) and sun-dried *B. alcinoe* [13], and boiled (30 min), sun-dried, and deep-fried (15–20 min) grasshoppers [50]. Recontamination from unhygienic processing and handling was associated with these observations. A recent investigation on the microbiological quality of processed edible insect products sold in Germany and the Netherlands coming from Europe or Asia also showed that the dried products did not comply with bacterial count recommendations for total bacterial counts, *Enterobacteriaceae*, staphylococci, bacilli, and yeast and mould, although the products were negative for *Salmonella*e, *L. monocytogenes*, *E. coli* and *Staphylococcus aureus* [54]. In a separate exploratory study, crickets (*Gryllus bimaculatus*) and superworms (*Zophobas atratus*) subjected to different combinations of cooking and drying regimes varied in the microbial counts, strongly displaying species- and treatment-specific patterns [55]. In the present study, the boiled or toasted samples subjected to oven-drying had lower TVC by about one order of magnitude compared to those subjected to toasting or boiling alone, whereas those subjected to solar-drying had a higher TVC by up to 1.6 orders of magnitude (Table 3), suggesting some recontamination during solar-drying. Nonetheless, the solar- or oven-drying techniques maintained the good microbiological quality of the boiled/toasted products. An increase in microbial counts was observed elsewhere for smoked crickets after subjection to drying [16].

Raw edible insects generally contain high numbers of mesophilic aerobes, bacterial endospores or spore-forming bacteria, *Enterobacteriaceae*, lactic acid bacteria, psychotropic aerobes, and fungi, and potentially harmful species may be present [56]. Pathogens comprising *Staphylococcus aureus*, *Bacillus cereus* and coliforms (including *E. coli*, and species of *Pseudomonas*, *Klebsiella*, *Aerobacter*, *Proteus*), *Salmonella* spp., *Listeria monocytogenes*, as well as moulds of the genera *Aspergillus*, *Penicillium*, and *Fusarium*, have been isolated [15,52,54]. The presence of *St. aureus* is significant because of the ability to produce enterotoxins, although the vegetative cells are destroyed by cooking, but easily reintroduced during handling [17]. *Pseudomonas* and *Proteus* spp. are proteolytic; sometimes lipolytic, and are therefore implicated in spoilage, undesirable flavour, and loss of nutritional value [52]. Some moulds of the genera *Aspergillus*, *Penicillium*, and *Fusarium* are mycotoxigenic [16,47]. Because bacterial populations are generally higher in the gut as compared to the skin, the destruction is sometimes not complete during processing if heat transfer to the inner tissues is inadequate [12]. Unhygienic handling, storage and retailing can also result in unsafe products [13]. Thus, two groups of microbiota should be of interest when assessing the microbial safety of insects—those that are intrinsically associated with insects and those that are introduced from the environment (rearing, handling, etc.). This study examined the decontamination dynamics of both reared and wild-harvested insects, and has shed some light on the potential variations in contamination when similar processes are applied for different insect species. However, a limitation of the study is that it did not extend the examinations to the broader microbial diversity within species, which should be of interest.

## 4. Conclusions

The objective of the present work was to evaluate the significance of some local processing methods on the nutritional and microbial quality of edible insects collected from the wild or reared for use in human or animal diets in East Africa. On dry matter basis, the nutritional value of all the processed products remained high but there were significant losses in fat and a subsequent gain in protein contents, especially when the insects were toasted or boiled. These effects have implications for the priorities of farmers who want to use insects as animal feed. Fish farmers may be more interested in protein-rich insects, whereas pig farmers may find high insect fat content desirable. Poultry farmers may desire insects with a high protein and high mineral content. The gain in fibre content when the insects are toasted is especially undesirable for fish, and could mean lower nutrient digestibility when ingested by humans or animals. The interaction of processing technique and insect species was significant; hence, the ultimate nutritional content is a function of insect species and processing method. Further studies should examine this interaction in the context of the qualitative aspects, such as changes in amino acid, fatty acid and micronutrient profiles, of the products. Insects can also contain functional substances that confer antimicrobial [11] and antioxidant activity [57], as well as anti-nutrients that might be affected by processing [58]. These were not investigated and should also form a basis for further investigations in the future. The interaction of processing technique and insect species was significant for microbiological quality. Boiling or toasting followed by drying in the oven were the most effective methods. The thermal processes destroyed active cells or lowered microbial load, and drying significantly lowered the water activity. To this end, further studies should examine shelf-life stability with respect to packaging and storage conditions.

## Figures and Tables

**Table 1:** Moisture, crude protein, and crude fat contents of raw and processed insects.

Insect Species				
	*H. illucens*	*A. domesticus*	*R. differens*	*S. littoralis*
Moisture (g/100g)				
Raw	67.5 ^Cb^	65.5 ^Ca^	65.4 ^Ca^	74.0 ^Cc^
Boiled	74.2 ^Db^ (+ 9.9)	71.3 ^Da^ (+8.9)	76.5 ^Dc^ (+17.0)	87.2 ^Dd^ (+17.8)
Toasted	32.7 ^Ba^ (−52.5)	44.2 ^Bb^ (−32.5)	46.1 ^Bb^ (−39.5)	32.6 ^Ba^ (−55.9)
Solar-dried	10.1 ^Ad^ (−85.0)	9.9 ^Ac^ (−84.9)	9.8^Ab^ (−85.0)	9.1 ^Aa^ (−87.7)
Oven-dried	8.9 ^Ac^ (−86.8)	8.8 ^Ac^ (−86.6)	8.3^Ab^ (−87.3)	7.2 ^Aa^ (−90.3)
Crude protein (g/100g DM)				
Raw	36.3 ^Aa^	52.3 ^Ad^	43.8 ^Ac^	38.5 ^Ab^
Boiled	39.6 ^Ca^ (+9.1)	57.5 ^Cd^ (+9.9)	48.8 ^Bc^ (+11.4)	41.9 ^Cb^ (+8.9)
Toasted	41.3 ^Da^ (+13.7)	59.5 ^Dd^ (+13.7)	53.5 ^Cc^ (+22.1)	43.8 ^Db^ (+13.7)
Solar-dried	37.0 ^Ba^ (+1.9)	53.1 ^ABd^ (+1.5)	42.3 ^Ac^ (+3.4)	39.7 ^Ab^ (+3.1)
Oven-dried	37.9 ^Ca^ (+4.4)	53.8 ^Bd^ (+2.9)	44.4 ^Ac^ (+1.4)	41.1 ^Bb^ (+7.1)
Crude fat (g/100g DM)				
Raw	29.6 ^Db^	18.3 ^Da^	28.4 ^Db^	17.4 ^Ca^
Boiled	20.3 ^Bb^ (−31.4)	16.5 ^Ba^ (−9.8)	24.9 ^Bc^ (−12.3)	16.3 ^Ba^ (−6.3)
Toasted	14.3 ^Ab^ (−51.7)	10.5 ^Aa^ (−42.6)	20.1 ^Ac^ (−29.2)	9.7 ^Aa^ (−44.3)
Solar-dried	28.2 ^Dc^ (−4.7)	17.9 ^CDa^ (−2.2)	27.5 ^CDb^ (−3.3)	17.3 ^Ca^ (−0.8)
Oven-dried	26.9 ^Cb^ (−9.2)	17.1 ^BCa^ (−6.6)	26.7 ^Cb^ (−6.1)	16.9 ^BCa^ (−2.9)

Means in the same column followed by the same capital letters, and means in the same row followed by the same small letters are not significantly different (*p* < 0.05; *n* = 3). Values in parentheses are the percent change relative to the raw product.

**Table 2:** Crude fibre, ash and carbohydrate contents of raw and processed insects.

Insect Species				
	*H. illucens*	*A. domesticus*	*R. differens*	*S. littoralis*
Crude Fibre (g/100g DM)				
Raw	8.6 ^Bc^	8.1 ^BCc^	4.2 ^Ba^	6.4 ^Bb^
Boiled	7.9 ^Ac^ (–8.1)	7.4 ^Ac^ (–8.6)	3.9 ^Aa^ (–7.1)	6.5 ^BCb^ (+1.6)
Toasted	10.6 ^Cd^ (+23.2)	8.2 ^Cc^ (+1.2)	4.5 ^Ca^ (+7.1)	6.2 ^Ab^ (–3.1)
Solar-dried	8.7 ^Bc^ (+1.2)	8.1 ^BCc^ (0.0)	4.2 ^Ba^ (0.0)	6.6 ^Cb^ (+3.1)
Oven-dried	8.5 ^Bc^ (–1.2)	8.0 ^Bc^ (–1.2)	4.1 ^Ba^ (–2.4)	6.6 ^Cb^ (+3.1)
Crude Ash (g/100g DM)				
Raw	3.9 ^Bb^	3.6 ^Bab^	3.1 ^Ba^	7.6 ^Bc^
Boiled	2.9 ^Aa^ (–25.6)	3.0 ^Aa^ (–16.7)	2.7 ^Aa^ (–12.9)	6.7 ^Ab^ (–11.8)
Toasted	4.3 ^Ca^ (+10.3)	4.2 ^Ca^ (+16.7)	4.3 ^Da^ (+38.7)	9.5 ^Cb^ (+25.0)
Solar-dried	3.8 ^Ba^ (–0.2.5)	3.6 ^Ba^ (0.0)	3.8 ^Ca^ (+22.5)	7.6 ^Bb^ (0.0)
Oven-dried	3.9 ^Ba^ (0.0)	3.5 ^Ba^ (–2.8)	3.7 ^Ca^ (+19.3)	7.7 ^Bb^ (+1.3)
Available Carbohydrate (g/100g DM)				
Raw	21.6 ^Ab^	17.7 ^Da^	20.4 ^Cb^	30.1 ^Cc^
Boiled	29.3 ^Cc^ (+35.6)	15.6 ^Aa^ (–11.9)	19.7 ^Bb^ (–3.4)	28.6 ^Bc^ (–4.9)
Toasted	29.5 ^Cb^ (+36.6)	17.6 ^CDa^ (–0.6)	17.7 ^Aa^ (–13.2)	30.7 ^Cb^ (+2.0)
Solar-dried	22.3 ^ABb^ (+3.2)	17.3 ^Ba^ (–2.3)	22.2 ^Eb^ (+8.8)	28.9 ^Bc^ (–4.0)
Oven-dried	22.8 ^Bb^ (+5.6)	17.5 ^BCa^ (–1.1)	21.2 ^Db^ (+3.9)	27.6 ^Ac^ (–8.3)

Means on the same column followed by the same capital letters, and means on the same row followed by the same small letters are not significantly different (*p* < 0.05; *n* = 3). Values in parentheses are the percent change relative to the raw product.

**Table 3:** Total viable counts and yeast and mould counts on raw and processed products.

	Insect Species			
	*H. illucens*	*A. domesticus*	*R. differens*	*S. littoralis*
Total viable count (Log CFU/g)				
Raw	7.7 ^Eb^	8.3 ^Fc^	9.1 ^Ed^	7.0 ^Ea^
Boiled	2.6 ^Bb^ (–66.2)	2.3 ^Ba^ (–72.3)	2.8 ^Bc^ (–69.2)	2.6 ^Bb^ (–62.9)
Toasted	2.8 ^BCa^ (–63.6)	3.1 ^Cd^ (–62.7)	2.9 ^Bb^ (–68.1)	3.0 ^BCc^ (–57.1)
Solar-dried	7.8 ^Eb^ (+1.3)	8.8 ^Fc^ (+6.0)	9.2 ^Ed^ (+1.1)	7.4 ^Ea^ (+5.7)
Oven-dried	6.2 ^Da^ (–19.4)	6.8 ^Eb^ (–18.1)	6.8 ^Db^ (–25.3)	6.4 ^Dc^ (–8.6)
Boiled + Solar-dried	3.1 ^Ca^ (–59.7)	3.9 ^Db^ (–53.0)	3.2 ^Ba^ (–64.8)	3.1 ^Ca^ (–55.7)
Toasted + Solar-dried	3.0 ^BCab^ (–61.0)	3.1 ^Cb^ (–62.6)	3.9 ^Cc^ (–57.1)	2.9 ^BC^ (–58.6)
Boiled + Oven-dried	1.6 ^Ab^ (–79.2)	1.0 ^Aa^ (–87.9)	1.8 ^Ac^ (80.2)	1.7 ^Abc^ (75.7)
Toasted + Oven-dried	1.8 ^Ab^ (–76.6)	1.3 ^Aa^ (–78.3)	2.0^Ac^ (–78.0)	2.1 ^Ac^ (–70.0)
Yeast and mould count (Log CFU/g)				
Raw	7.6 ^Cb^	7.8 ^Cb^	8.2 ^Cc^	6.4 ^Ba^
Boiled	0.0	0.0	0.0	0.0
Toasted	0.0	0.0	0.0	0.0
Solar-dried	6.5 ^Ba^ (–14.5)	7.0 ^Bb^ (–10.2)	8.0 ^Bc^ (–2.4)	6.3 ^Ba^ (–1.5)
Oven-dried	4.2 ^Ab^ (–44.7)	3.2 ^Aa^ (–59.0)	5.5 ^Ac^ (–32.9)	3.0 ^Aa^ (–53.1)
Boiled + Solar-dried	0.0	0.0	0.0	0.0
Toasted + Solar-dried	0.0	0.0	0.0	0.0
Boiled + Oven-dried	0.0	0.0	0.0	0.0
Toasted + Oven-dried	0.0	0.0	0.0	0.0

Means on the same column followed by the same capital letters, and means on the same row followed by the same small letters are not significantly different (*p* < 0.05; *n* = 3). Values in parentheses are the percent change relative to the raw product.

**Table 4:** Counts of Lac+ enteric bacteria (Log colony-forming units (CFU)/g) on the raw insects and the processed products.

	Insect Species			
	*H. illucens*	*A. domesticus*	*R. differens*	*S. littoralis*
Raw	6.4 ^Db^	6.1 ^Cb^	5.9 ^Cb^	0.0 ^Aa^
Boiled	0.0	0.0	0.0	0.0
Toasted	0.0	0.0	0.0	0.0
Solar-dried	2.1 ^Cb^ (−67.2)	2.8 ^Bc^ (−54.1)	2.3 ^Bb^ (−60.1)	0.0 ^Aa^
Oven dried	1.1 ^Bb^ (−82.8)	1.5 ^Bc^ (−75.4)	1.0 ^Bb^ (−83.1)	0.0 ^Aa^
Boiled + Solar-dried	0.0	0.0	0.0	0.0
Toasted + Solar-dried	0.0	0.0	0.0	0.0
Boiled + Oven-dried	0.0	0.0	0.0	0.0
Toasted + Oven-dried	0.0	0.0	0.0	0.0

Means in the same column followed by the same capital letters, and means on the same row followed by the same small letters are not significantly different (*p* < 0.05; *n* = 3). Values in parentheses are the percent change relative to the raw product.

**Table 5:** Counts of *St. aureus,* and presence (+) or absence (−) of *Salmonella* on the raw and processed products.

	Insect Species			
	*H. illucens*	*A. domesticus*	*R. differens*	*S. littoralis*
*St. aureus* (Log CFU/g)				
Raw	7.9 ^Fa^	8.3 ^Fb^	9.1 ^Ec^	7.7 ^Ea^
Boiled	2.5 ^Db^ (−68.4)	2.1 ^CDa^ (−74.7)	3.3 ^Bd^ (−63.7)	2.9 ^Cc^ (−62.3)
Toasted	1.5 ^Ba^ (−80.1)	1.9 ^Cb^ (−77.1)	2.9 ^Bc^ (−68.1)	1.8 ^Ab^ (−76.6)
Solar-dried	3.0 ^Ea^ (−62.0)	2.9 ^Ea^ (−65.1)	4.5 ^Dc^ (−50.5)	3.8 ^Db^ (−50.6)
Oven-dried	1.7 ^BCa^ (−78.5)	1.6 ^BCa^ (−80.7)	3.9 ^Cc^ (−57.1)	3.2 ^Cb^ (−58.4)
Boiled + Solar-dried	2.6 ^Da^ (−67.1)	3.0 ^Eb^ (−63.8)	4.0 ^Cc^ (−56.0)	3.1 ^Cb^ (−59.7)
Toasted + Solar-dried	2.0 ^Ca^ (−74.9)	2.4 ^Db^ (−71.1)	3.1 ^Bc^ (−65.9)	2.1 ^Ba^ (−72.7)
Boiled + Oven-dried	1.2 ^ABa^ (−8.8)	1.3 ^ABa^ (−84.3)	2.1 ^Ac^ (−76.9)	1.9 ^ABb^ (−75.3)
Toasted + Oven-dried	0.9 ^Aa^ (−88.6)	1.0 ^Aa^ (−87.9)	2.0 ^Ac^ (−78.0)	1.6 ^Ab^ (−79.2)
*Salmonella*				
Raw	+	+	+	+
Boiled	−	−	−	−
Toasted	−	−	−	−
Solar-dried	+	+	+	+
Oven-dried	+	+	+	+
Boiled + Solar-dried	−	−	−	−
Toasted + Solar-dried	−	−	−	−
Boiled + Oven-dried	−	−	−	−
Toasted + Oven-dried	−	−	−	−

For *St. aureus*, means in the same column followed by the same capital letters, and means in the same row followed by the same small letters are not significantly different (*p* < 0.05; *n* = 3). Values in parentheses are the percent change relative to the raw product.

## Supplementary Materials

The following are available as https://www.mdpi.com/2304-8158/9/5/574/s1, supplementary material S1: Traditional processing of edible insects in Africa; supplementary material S2: ANOVA tables and graphical representation of the interaction effects of insect species and processing technique on proximate composition parameters; supplementary material S3: ANOVA tables and graphical representation of interaction effects on microbiological quality parameters.
