# Supplementary material for: Effects of Traditional Processing Techniques on the Nutritional and Microbiological Quality of Four Edible Insect Species Used for Food and Feed in East Africa
Source: Foods. 2020 May 4;9(5):574. doi: 10.3390/foods9050574 (PMC7278588; doi:10.3390/foods9050574)
Supplement: Supplementary file 1 [file foods-09-00574-s001.pdf]

Supplementary Materials

S1: Traditional processing of edible insects in Africa

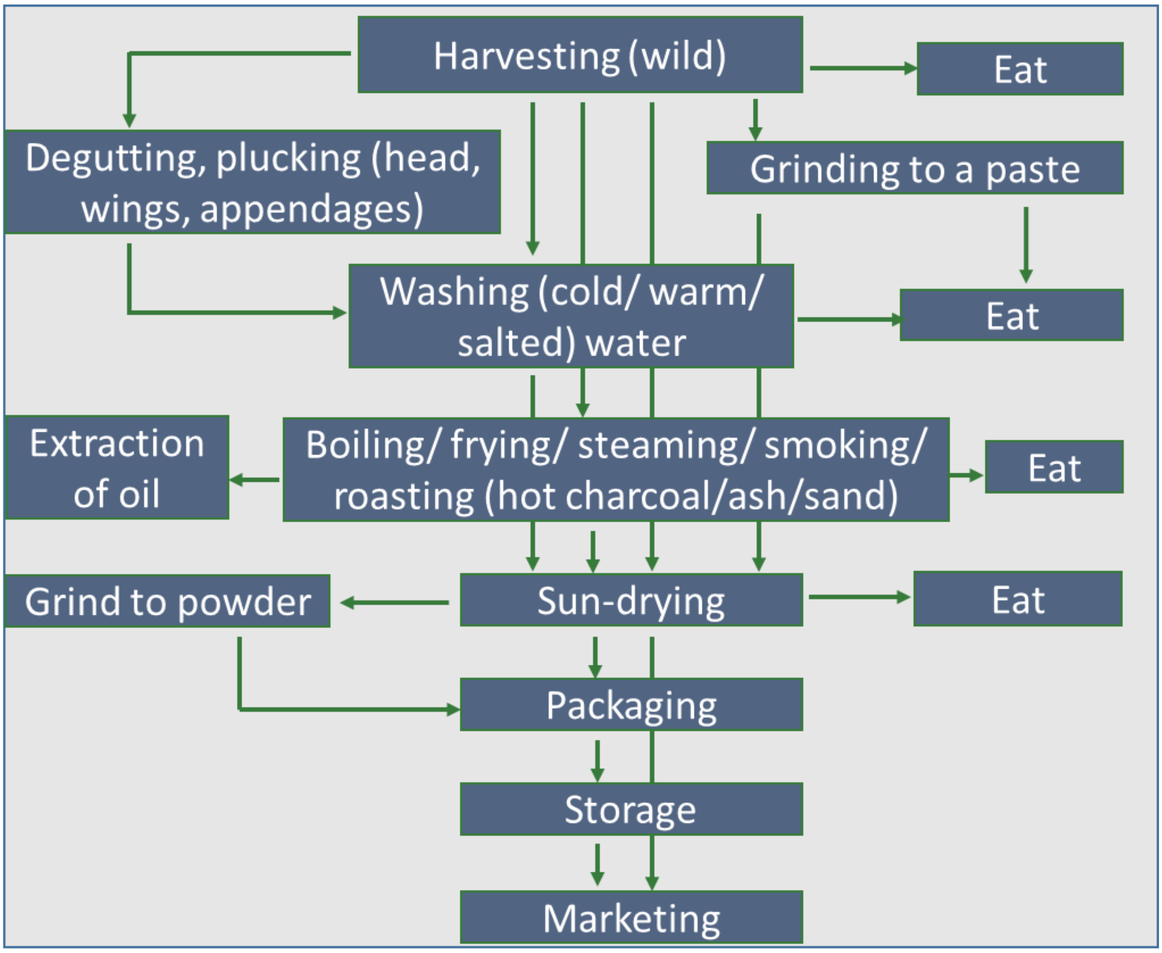

Figure S1.1: Traditional processing of edible insects in Africa (Extracted from: Mutungi, C.; Irungu, F.G.; Nduko, J.; Mutua, F.; Affognon, H.; Nakimbugwe, D.; Ekesi, S.; Fiaboe, K.K.M. Postharvest processes of edible insects in Africa: A review of processing methods, and the implications for nutrition, safety and new products development. Critical Reviews in Food Science and Nutrition **2019**, 59, 276-298, doi:10.1080/10408398.2017.1365330)

S2: Analysis of variance (ANOVA) tables and graphical representations of the interaction effects of insect species and processing technique on proximate composition parameters

Table S2.1: Effect of processing method and species on moisture content

Tests of Between-Subjects Effects
Dependent Variable: Moisture

Source	Type III Sum of Squares	df	Mean Square	F	Sig.
Corrected Model	50340.267[a]	19	2649.488	2890.350	.000
Intercept	98253.067	1	98253.067	107185.164	.000
Insect_spp	90.400	3	30.133	32.873	.000
Processing_method	49306.433	4	12326.608	13447.209	.000
Insect_spp * Processing_method	943.433	12	78.619	85.767	.000
Error	36.667	40	.917		
Total	148630.000	60			
Corrected Total	50376.933	59			

a. R Squared = .999 (Adjusted R Squared = .999)

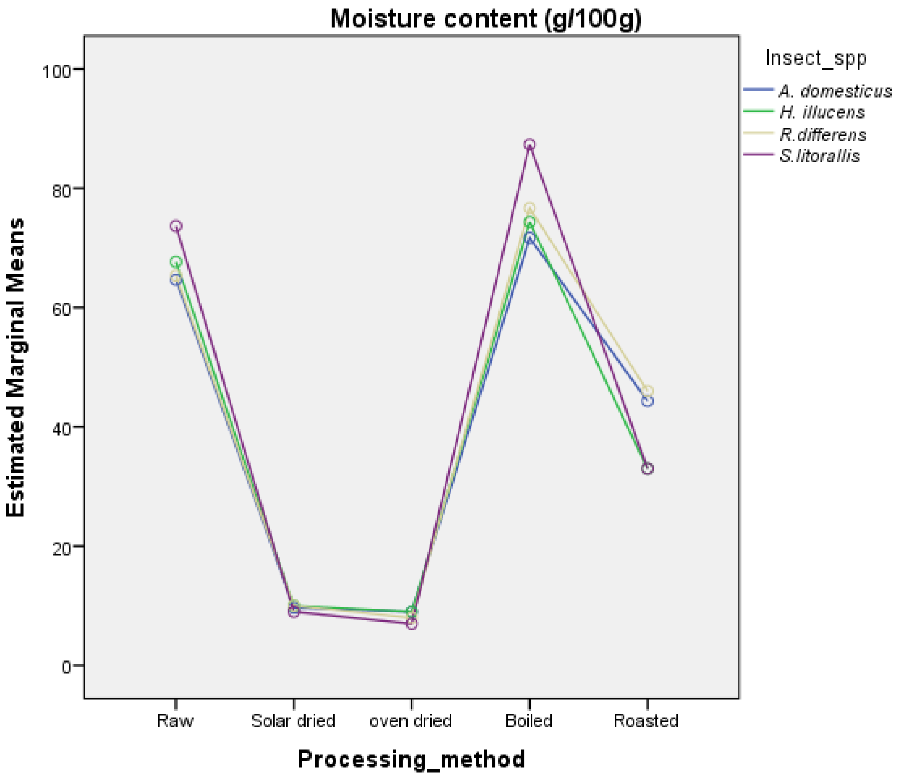

Figure S2.1: Interaction effect of insect species and processing technique on moisture content

Table S2.2: Effect of processing method and species on crude protein content

Tests of Between-Subjects Effects

Dependent Variable: %Protein_dm

Source	Type III Sum of Squares	df	Mean Square	F	Sig.
Corrected Model	2919.267[a]	19	153.646	288.086	.000
Intercept	123125.400	1	123125.400	230860.125	.000
Insect_spp	2459.800	3	819.933	1537.375	.000
Processing_method	399.100	4	99.775	187.078	.000
Insect_spp * Processing_method	60.367	12	5.031	9.432	.000
Error	21.333	40	.533		
Total	126066.000	60			
Corrected Total	2940.600	59			

a. R Squared = .993 (Adjusted R Squared = .989)

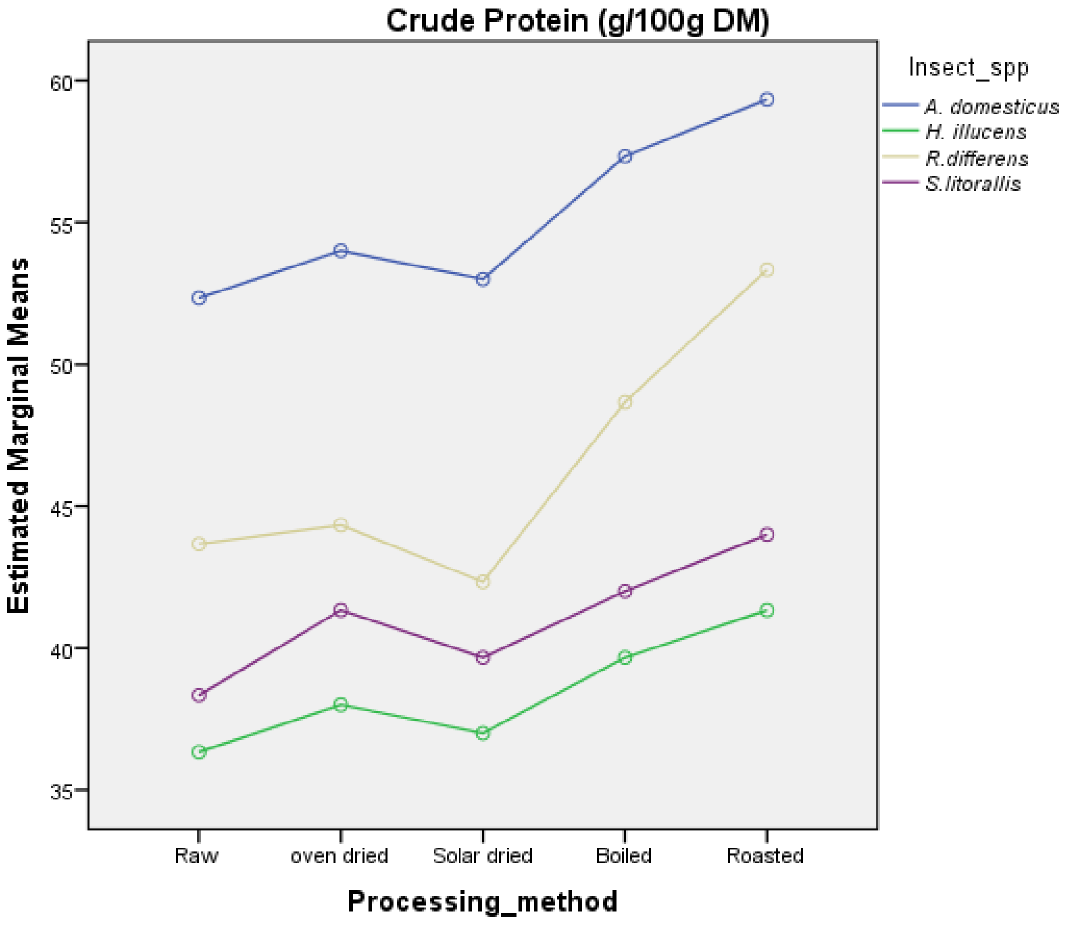

Figure S2.2: Interaction effect of insect species and processing technique on protein content

Table S2.3: Effect of processing method and species on crude fat content

Tests of Between-Subjects Effects

Dependent Variable: %Fat_dm

Source	Type III Sum of Squares	df	Mean Square	F	Sig.
Corrected Model	2100.400[a]	19	110.547	473.774	.000
Intercept	24644.267	1	24644.267	105618.286	.000
Insect_spp	1218.400	3	406.133	1740.571	.000
Processing_method	760.567	4	190.142	814.893	.000
Insect_spp * Processing_method	121.433	12	10.119	43.369	.000
Error	9.333	40	.233		
Total	26754.000	60			
Corrected Total	2109.733	59			

a. R Squared = .996 (Adjusted R Squared = .993)

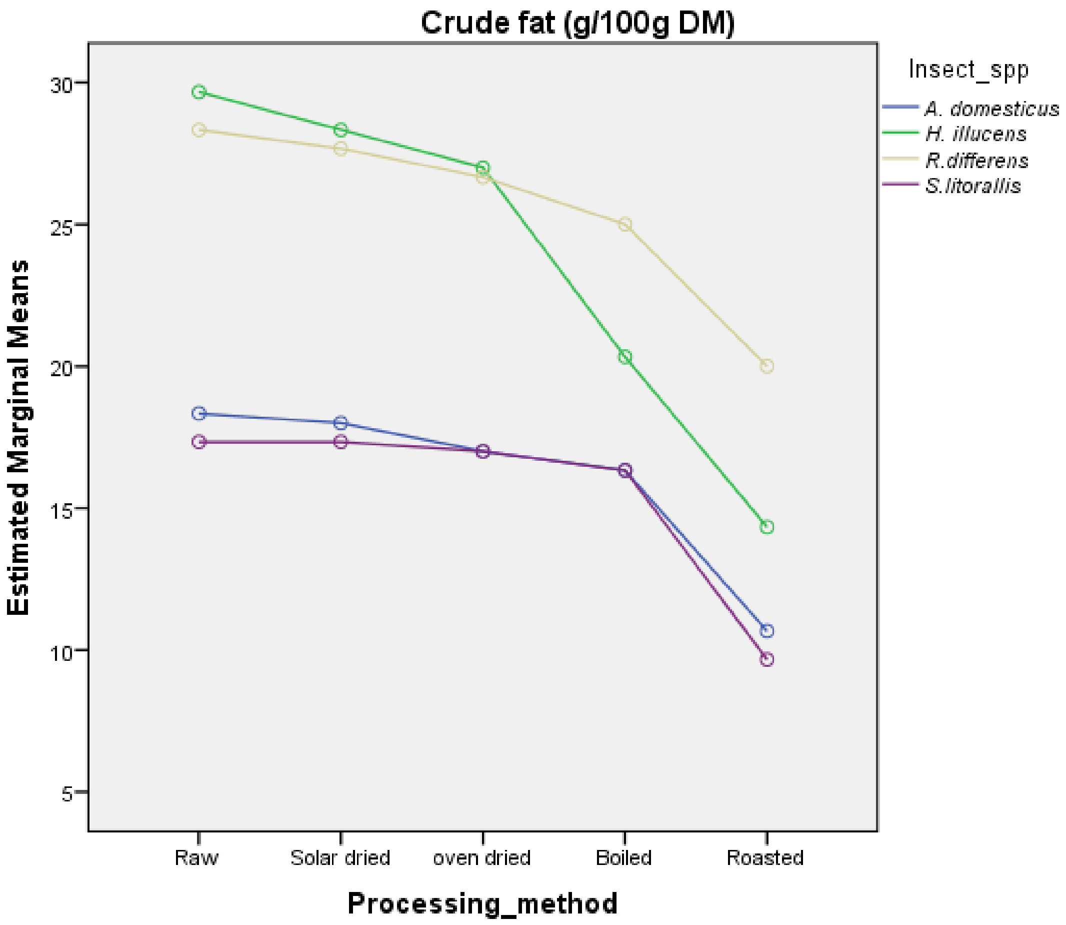

Figure S2.3: Interaction effect of insect species and processing technique on crude fat content

Table S2.4: Effect of processing method and species on crude fibre content

Tests of Between-Subjects Effects
Dependent Variable: %Fiber_dm

Source	Type III Sum of Squares	df	Mean Square	F	Sig.
Corrected Model	206.850[a]	19	10.887	81.651	.000
Intercept	2842.817	1	2842.817	21321.125	.000
Insect_spp	189.650	3	63.217	474.125	.000
Processing_method	5.433	4	1.358	10.188	.000
Insect_spp * Processing_method	11.767	12	.981	7.354	.000
Error	5.333	40	.133		
Total	3055.000	60			
Corrected Total	212.183	59			

a. R Squared = .975 (Adjusted R Squared = .963)

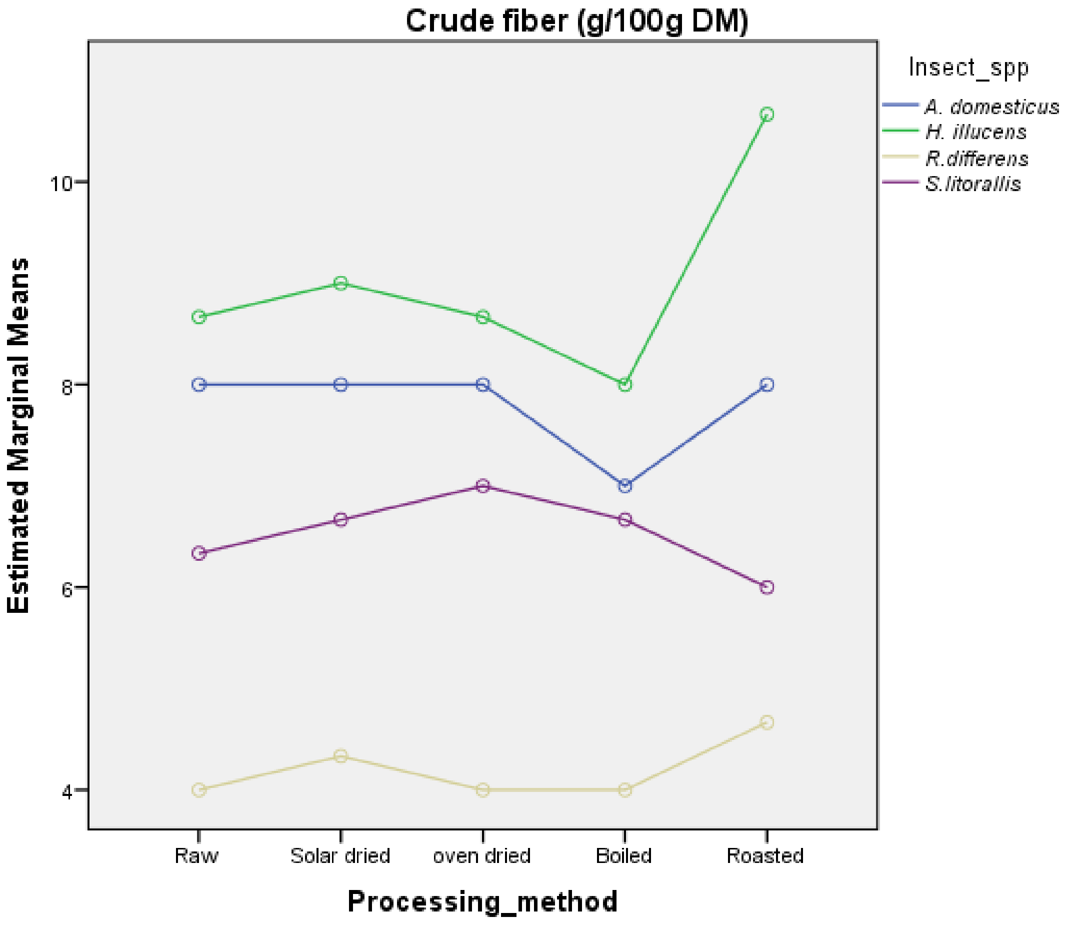

Figure S2.4: Interaction effect of insect species and processing technique on crude fibre content

Table S2.5: Effect of processing method and species on crude ash content

Tests of Between-Subjects Effects

Dependent Variable: %Ash_dm

Source	Type III Sum of Squares	df	Mean Square	F	Sig.
Corrected Model	233.600[a]	19	12.295	81.965	.000
Intercept	1382.400	1	1382.400	9216.000	.000
Insect_spp	205.333	3	68.444	456.296	.000
Processing_method	20.433	4	5.108	34.056	.000
Insect_spp * Processing_method	7.833	12	.653	4.352	.000
Error	6.000	40	.150		
Total	1622.000	60			
Corrected Total	239.600	59			

a. R Squared = .975 (Adjusted R Squared = .963)

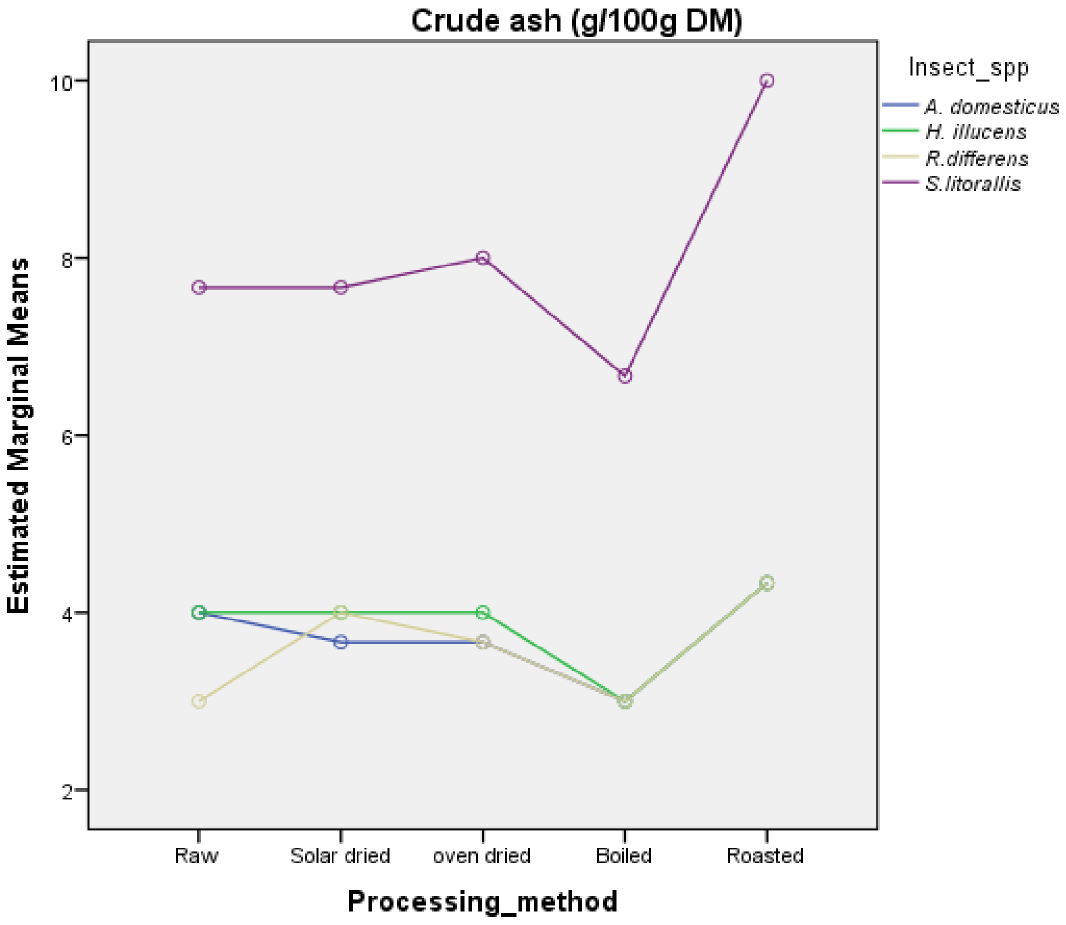

Figure S2.5: Interaction effect of insect species and processing technique on crude ash content

Table S2.6: Effect of processing method and species on available carbohydrate content

Tests of Between-Subjects Effects

Dependent Variable: %CHO_dm

Source	Type III Sum of Squares	df	Mean Square	F	Sig.
Corrected Model	1514.933[a]	19	79.733	93.804	.000
Intercept	31373.067	1	31373.067	36909.490	.000
Insect_spp	1271.733	3	423.911	498.719	.000
Processing_method	20.933	4	5.233	6.157	.001
Insect_spp * Processing_method	222.267	12	18.522	21.791	.000
Error	34.000	40	.850		
Total	32922.000	60			
Corrected Total	1548.933	59			

a. R Squared = .978 (Adjusted R Squared = .968)

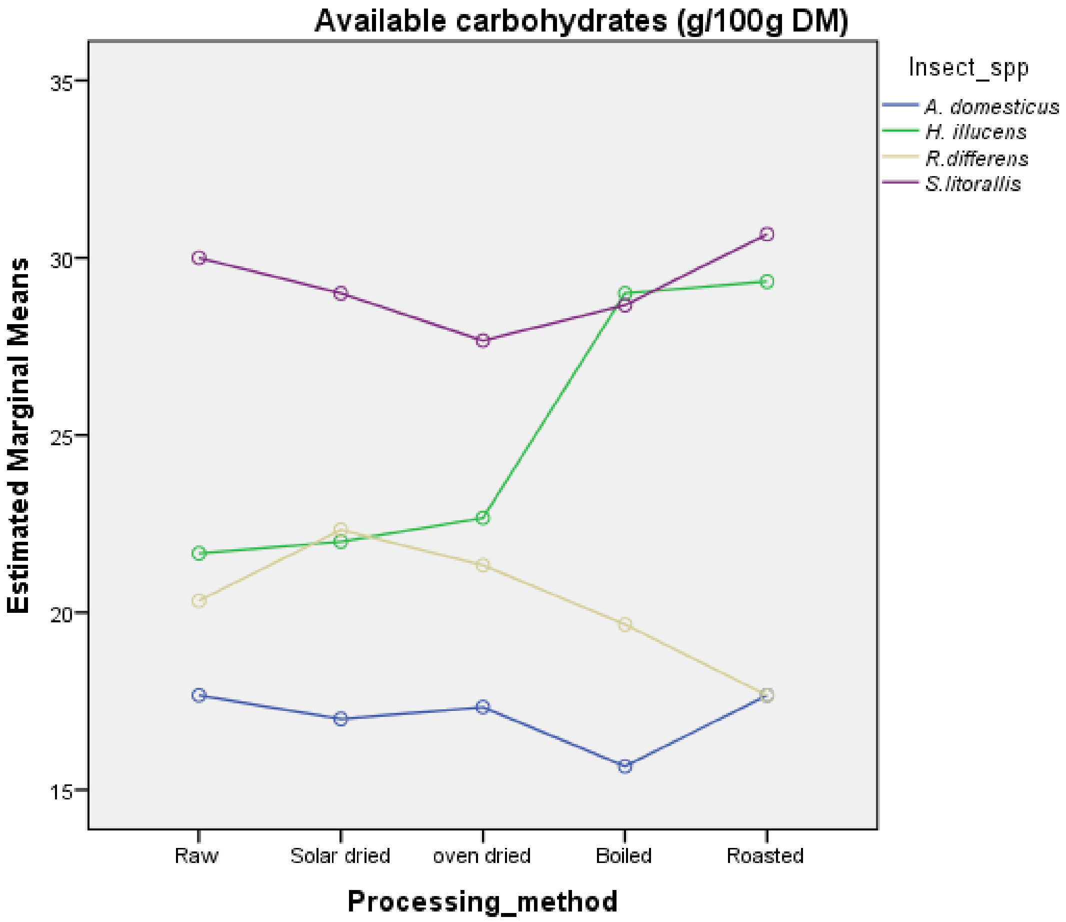

Figure S2.6: Interaction effect of insect species and processing technique on carbohydrate content

S3. Anova tables and graphical representation of the interaction effects of insect species and processing technique on microbiological quality parameters

Table S3.1: Effect of processing method and species on TVC (Log CFU/g)

Tests of Between-Subjects Effects
Dependent Variable: TVC

Source	Type III Sum of Squares	df	Mean Square	F	Sig.
Corrected Model	682.769[a]	35	19.508	421.366	.000
Intercept	1984.898	1	1984.898	42873.800	.000
Insect_spp	5.657	3	1.886	40.733	.000
Processing_method	656.352	8	82.044	1772.150	.000
Insect_spp * Processing_method	20.759	24	.865	18.683	.000
Error	3.333	72	.046		
Total	2671.000	108			
Corrected Total	686.102	107			

a. R Squared = .995 (Adjusted R Squared = .993)

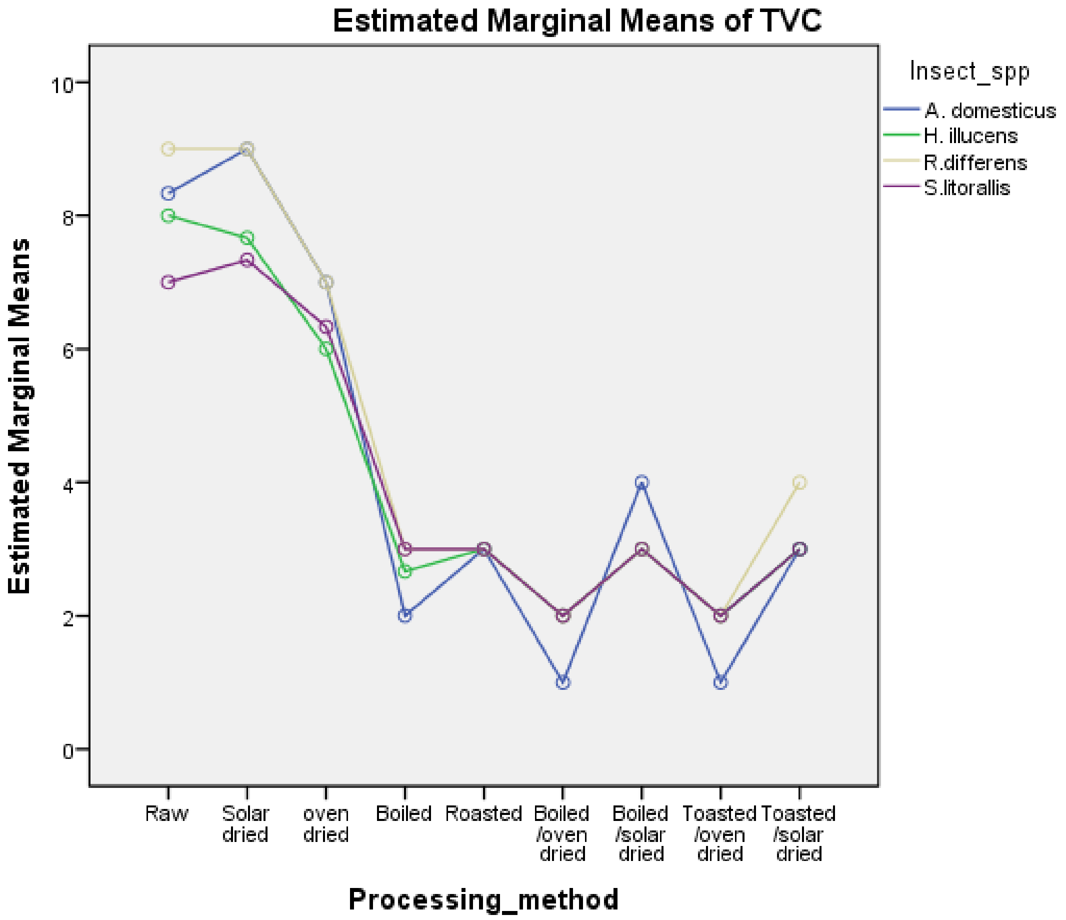

Figure S3.1: Interaction effect of insect species and processing technique on TVC

Table S3.2: Effect of processing method and species on Enterobacteriaceae (Log CFU/g)

Tests of Between-Subjects Effects

Dependent Variable: Enterobacteria

Source	Type III Sum of Squares	df	Mean Square	F	Sig.
Corrected Model	773.583[a]	35	22.102	1193.529	.000
Intercept	200.083	1	200.083	10804.500	.000
Insect_spp	2.102	3	.701	37.833	.000
Processing_method	754.667	8	94.333	5094.000	.000
Insect_spp * Processing_method	16.815	24	.701	37.833	.000
Error	1.333	72	.019		
Total	975.000	108			
Corrected Total	774.917	107			

a. R Squared = .998 (Adjusted R Squared = .997)

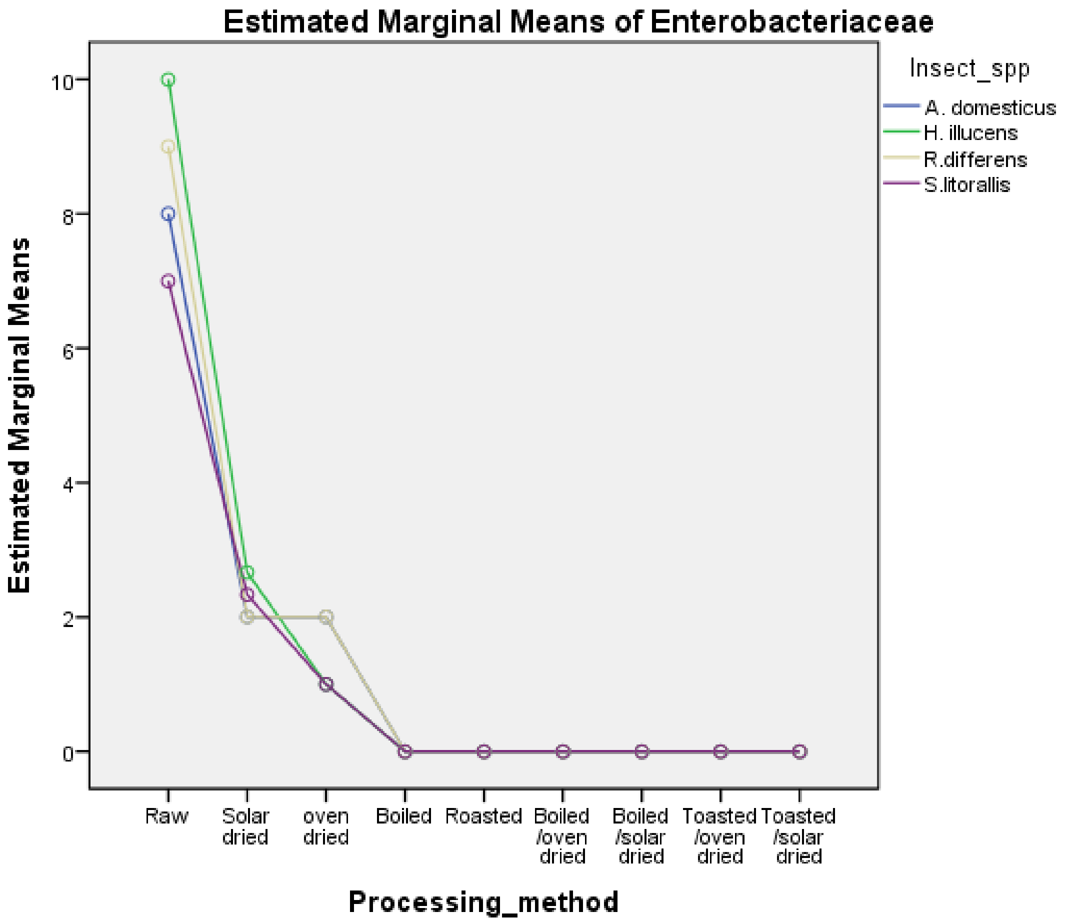

Figure S3.2: Interaction effect of insect species and processing technique on Enterobacteriaceae

Table S3.3: Effect of processing method and species on YMC (Log CFU/g)

Tests of Between-Subjects Effects
Dependent Variable: Yeast_molds

Source	Type III Sum of Squares	df	Mean Square	F	Sig.
Corrected Model	1014.519[a]	35	28.986	1565.257	.000
Intercept	448.148	1	448.148	24200.000	.000
Insect_spp	7.481	3	2.494	134.667	.000
Processing_method	985.019	8	123.127	6648.875	.000
Insect_spp * Processing_method	22.019	24	.917	49.542	.000
Error	1.333	72	.019		
Total	1464.000	108			
Corrected Total	1015.852	107			

a. R Squared = .999 (Adjusted R Squared = .998)

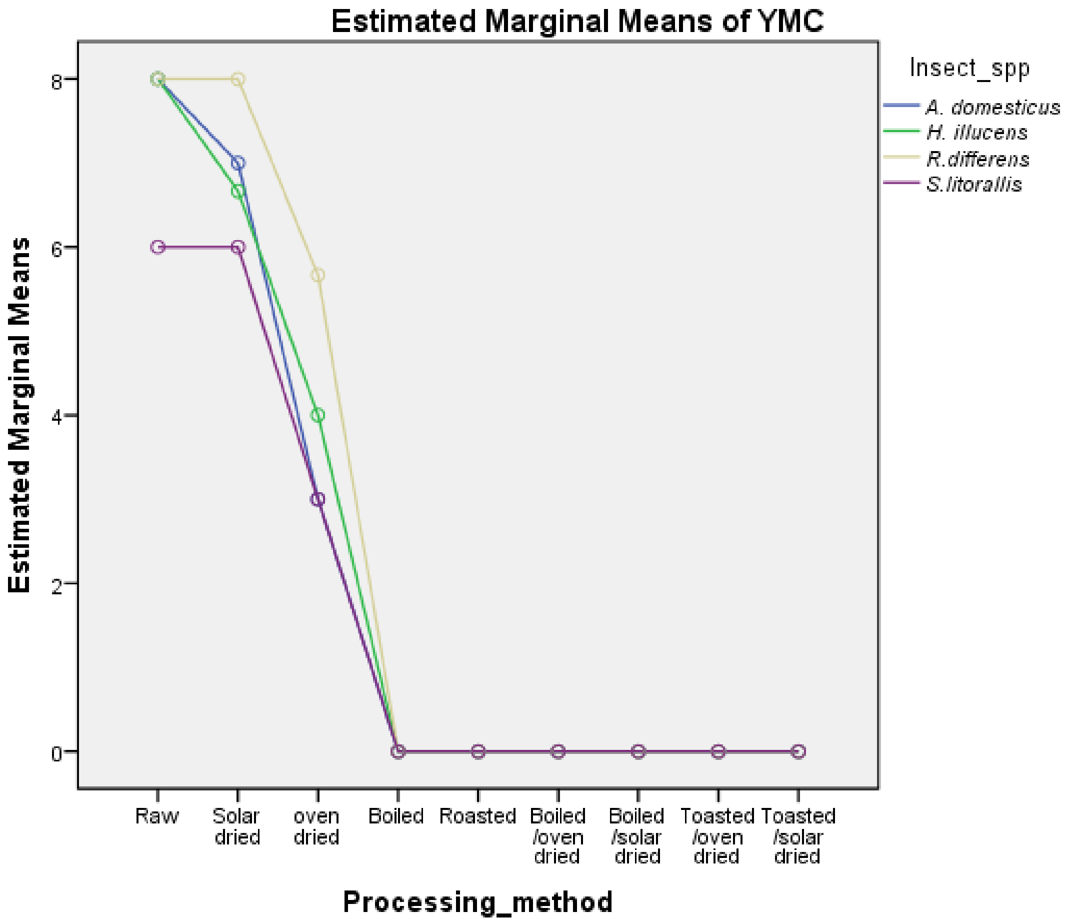

Figure S3.3: Interaction effect of insect species and processing technique on YMC

Table S3.4: Effect of processing method and species on coliform count (Log CFU/g)

Tests of Between-Subjects Effects

Dependent Variable: coliform count

Source	Type III Sum of Squares	df	Mean Square	F	Sig.
Corrected Model	876.333[a]	35	25.038	1352.057	.000
Intercept	320.333	1	320.333	17298.000	.000
Insect_spp	9.296	3	3.099	167.333	.000
Processing_method	836.833	8	104.604	5648.625	.000
Insect_spp * Processing_method	30.204	24	1.258	67.958	.000
Error	1.333	72	.019		
Total	1198.000	108			
Corrected Total	877.667	107			

a. R Squared = .998 (Adjusted R Squared = .998)

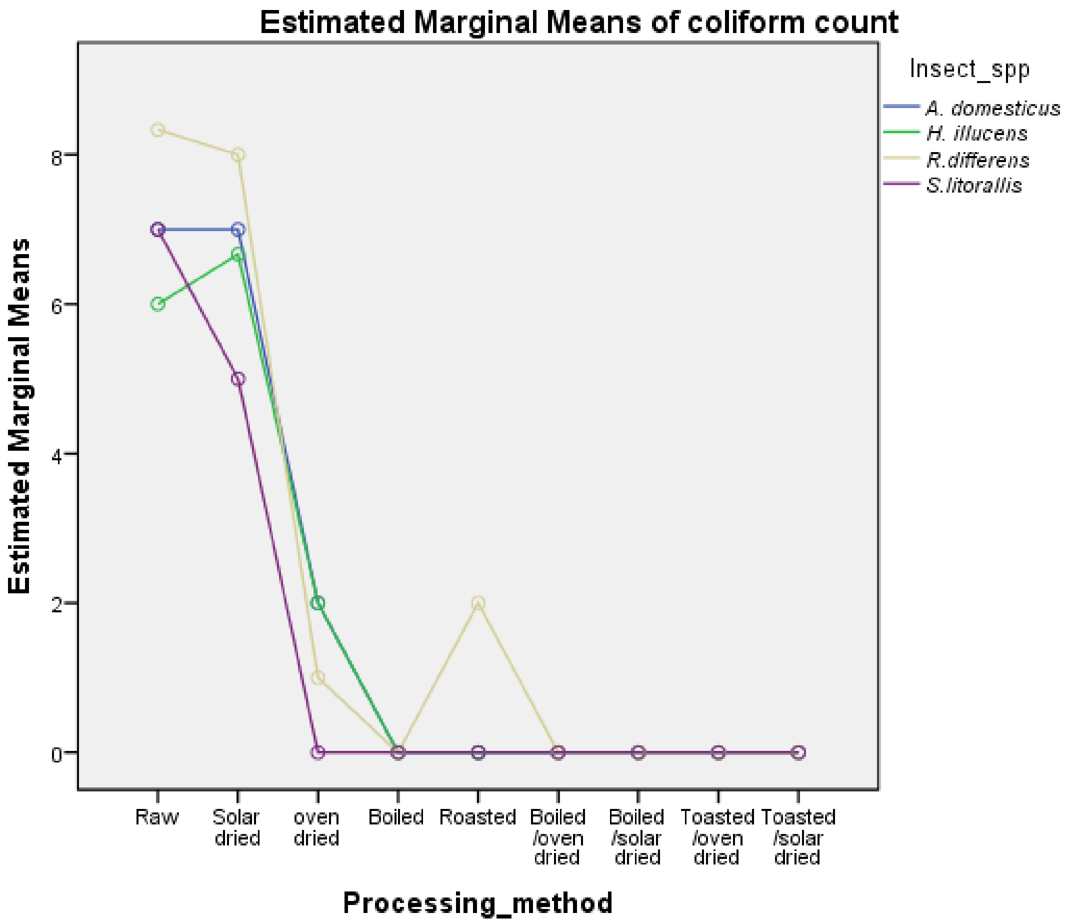

Figure S3.4: Interaction effect of insect species and processing technique on coliform count

Table S3.5: Effect of processing method and species on lactose positive enteric bacteria (Log CFU/g)

Tests of Between-Subjects Effects

Dependent Variable: Lactose positive enteric bacteria

Source	Type III Sum of Squares	df	Mean Square	F	Sig.
Corrected Model	320.852[a]	35	9.167	990.057	.000
Intercept	68.481	1	68.481	7396.000	.000
Insect_spp	23.444	3	7.815	844.000	.000
Processing_method	221.352	8	27.669	2988.250	.000
Insect_spp * Processing_method	76.056	24	3.169	342.250	.000
Error	.667	72	.009		
Total	390.000	108			
Corrected Total	321.519	107			

a. R Squared = .998 (Adjusted R Squared = .997)

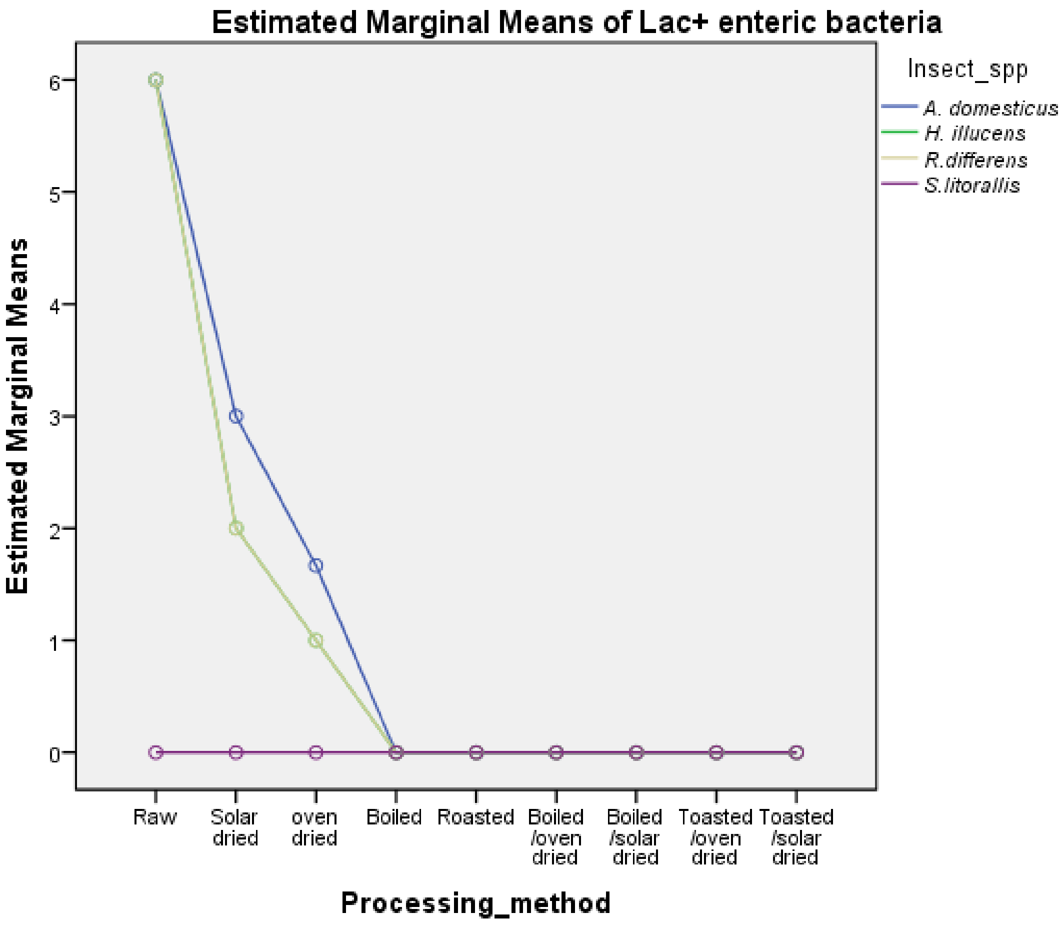

Figure S3.5: Interaction effect of insect species and processing technique on Lac+ enteric bacteria

Table S3.6: Effect of processing method and insect species on S. aureus (Log CFU/g)

Tests of Between-Subjects Effects

Dependent Variable: S_aureus

Source	Type III Sum of Squares	df	Mean Square	F	Sig.
Corrected Model	477.333[a]	35	13.638	736.457	.000
Intercept	1045.333	1	1045.333	56448.000	.000
Insect_spp	29.852	3	9.951	537.333	.000
Processing_method	439.333	8	54.917	2965.500	.000
Insect_spp * Processing_method	8.148	24	.340	18.333	.000
Error	1.333	72	.019		
Total	1524.000	108			
Corrected Total	478.667	107			

a. R Squared = .997 (Adjusted R Squared = .996)

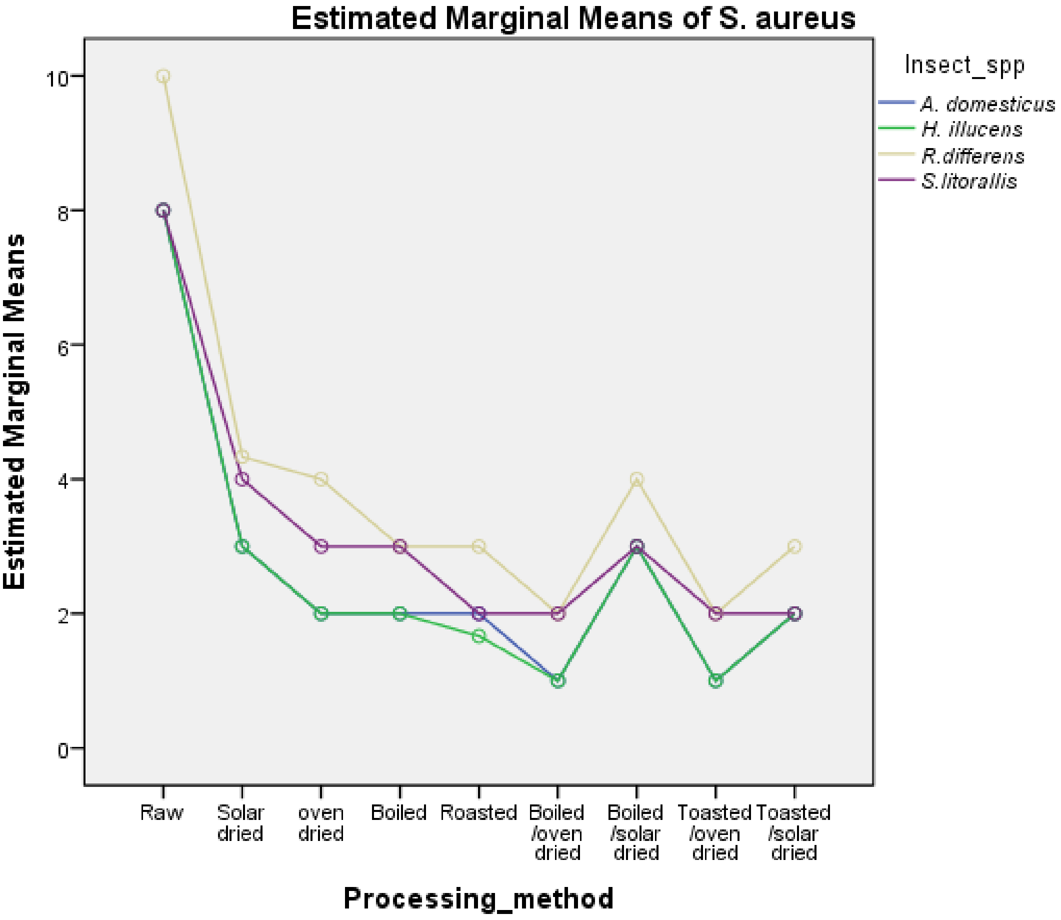

Figure S3.6: Interaction effect of insect species and processing technique on Staphylococcus aureus
